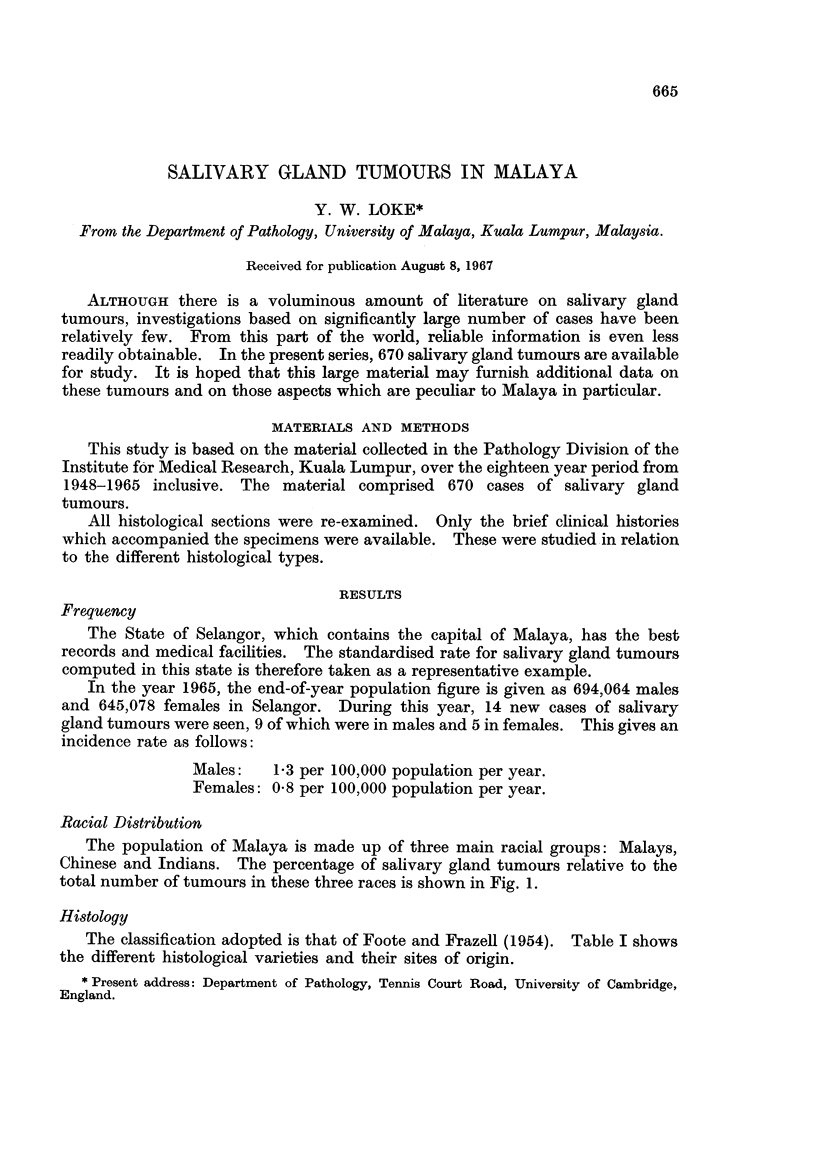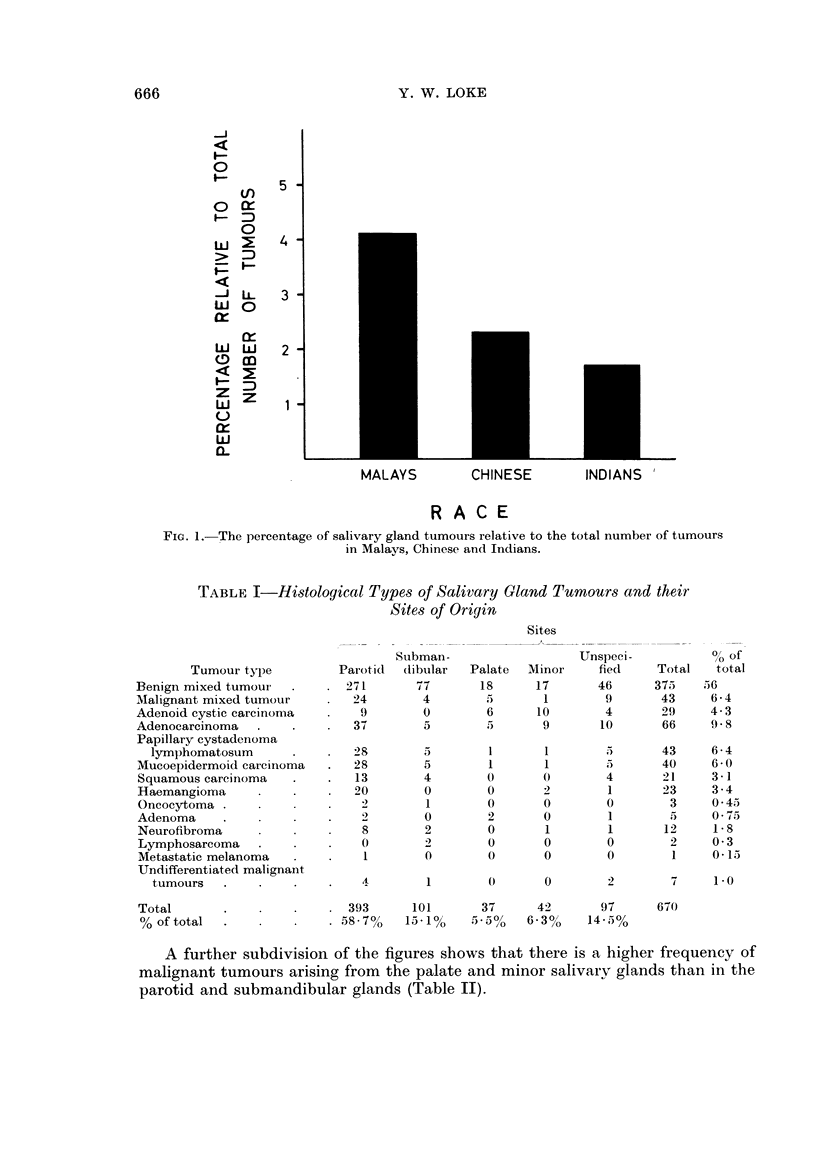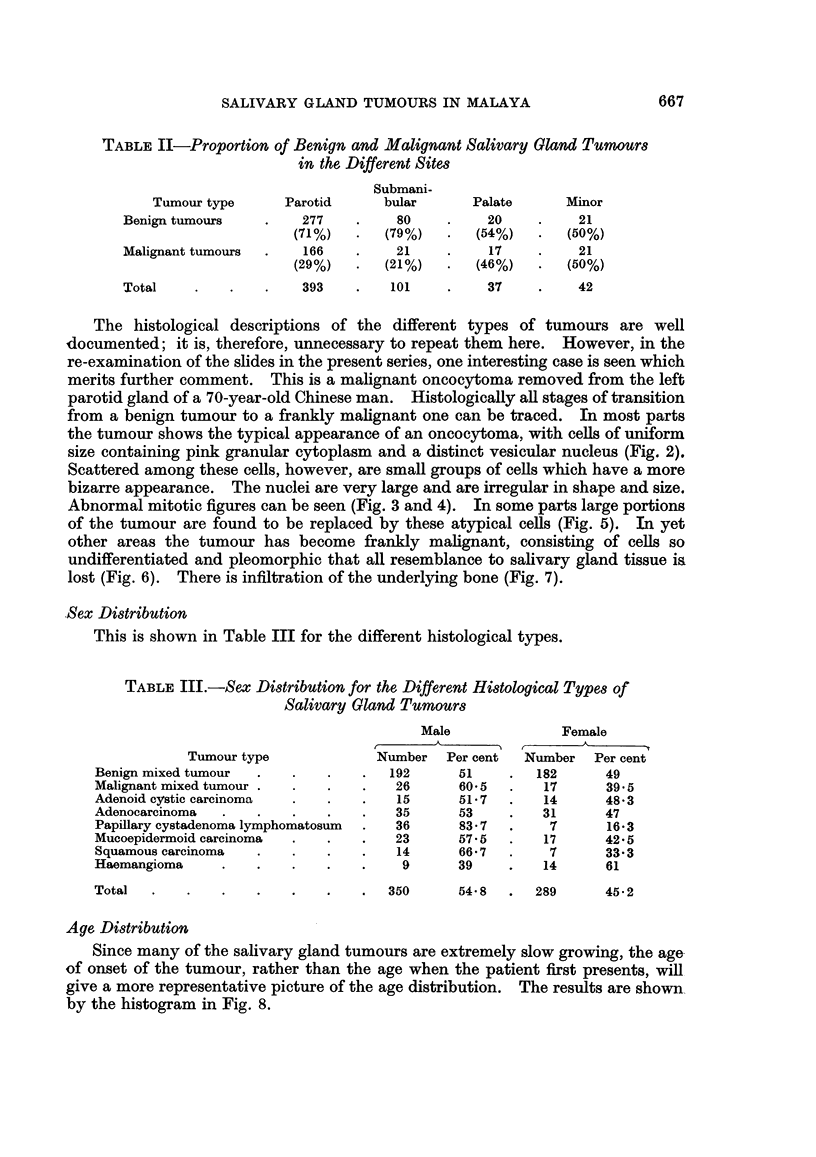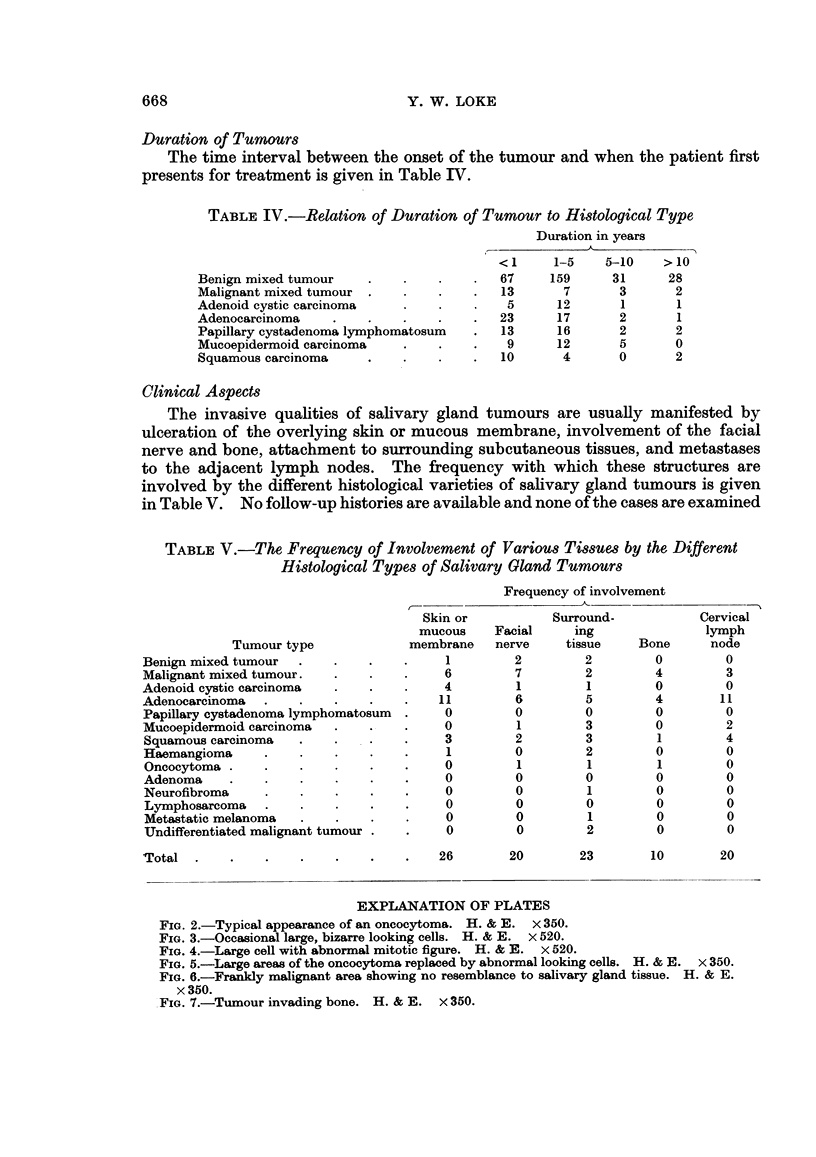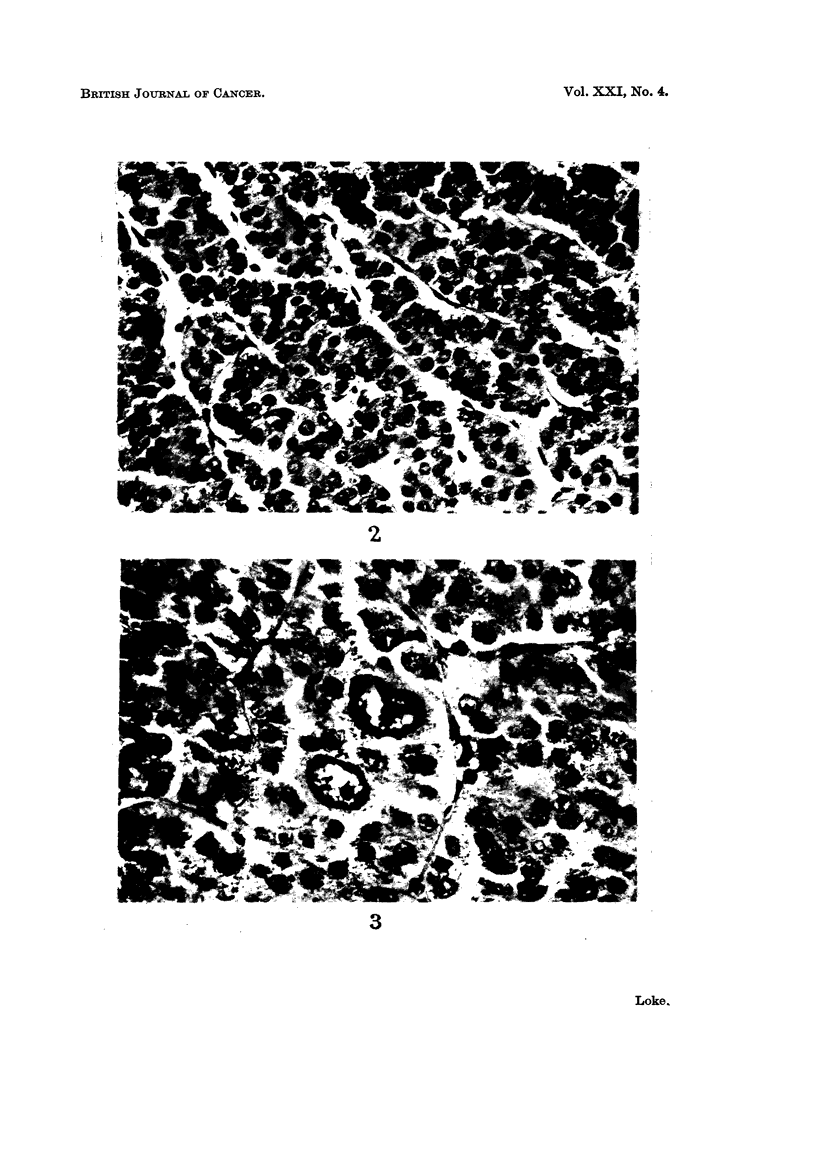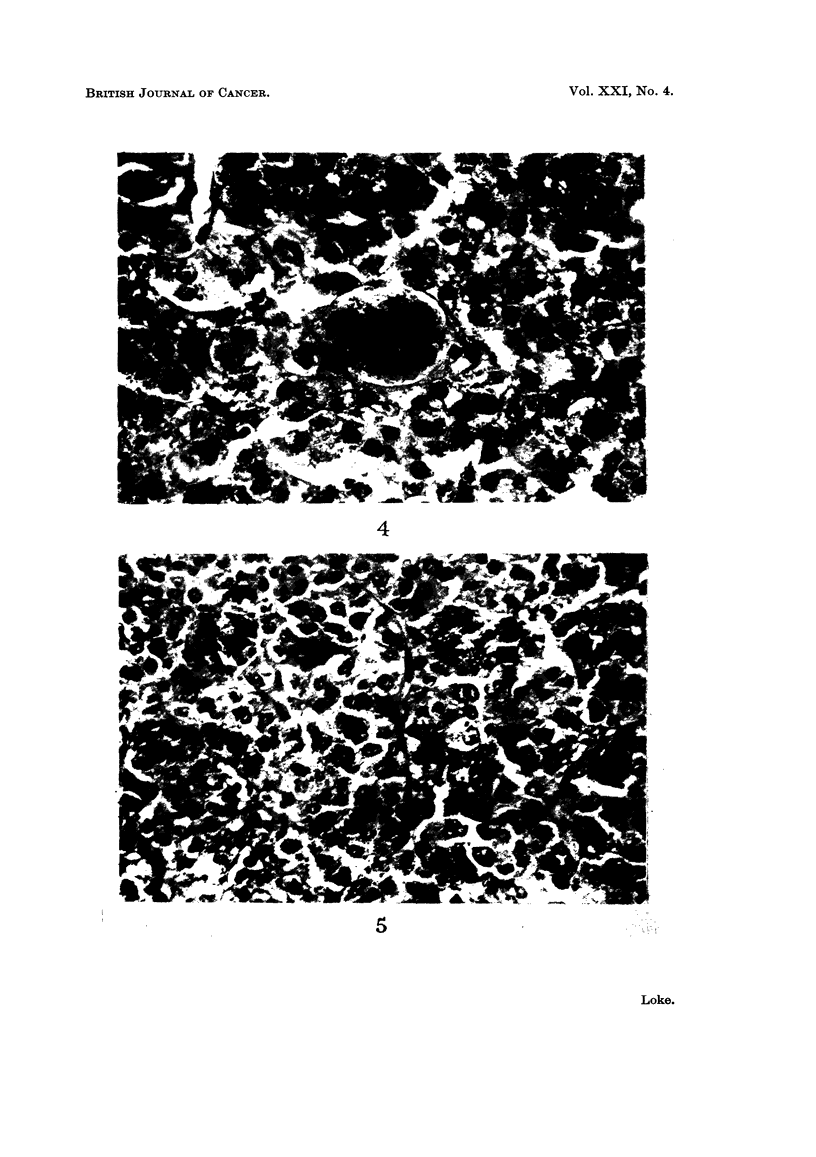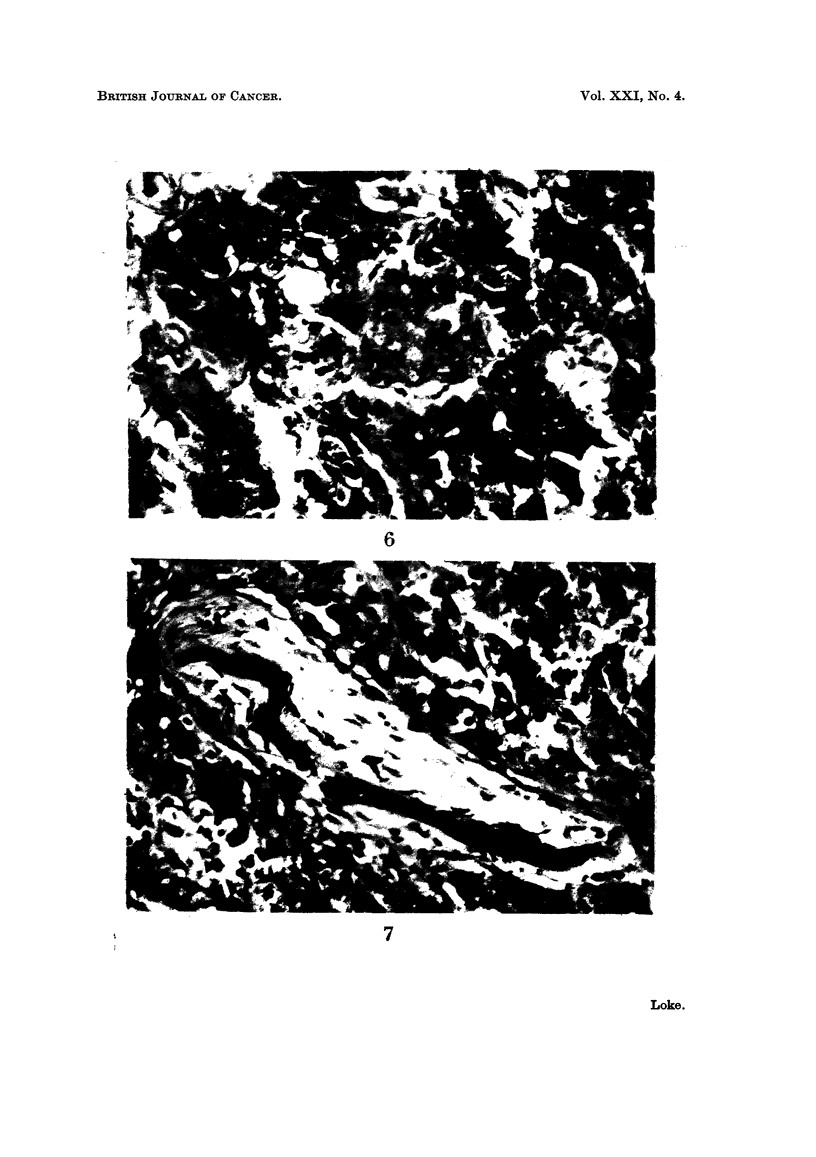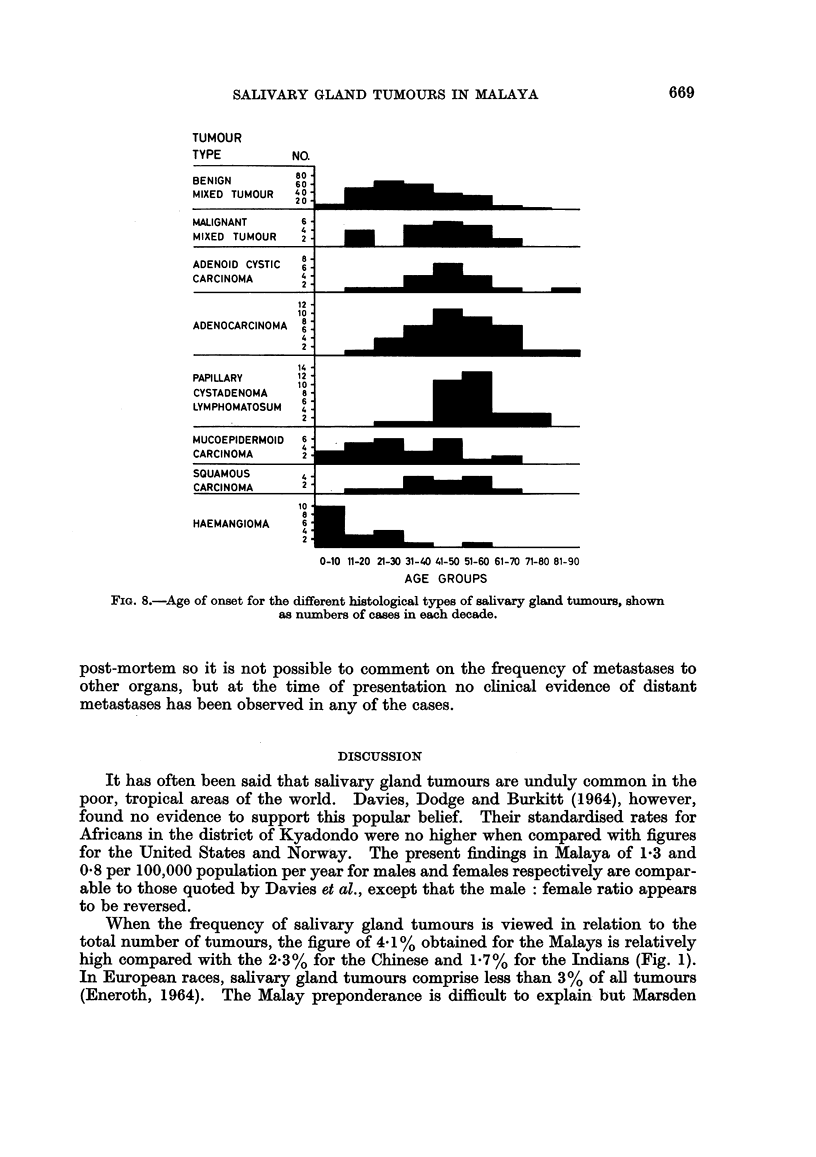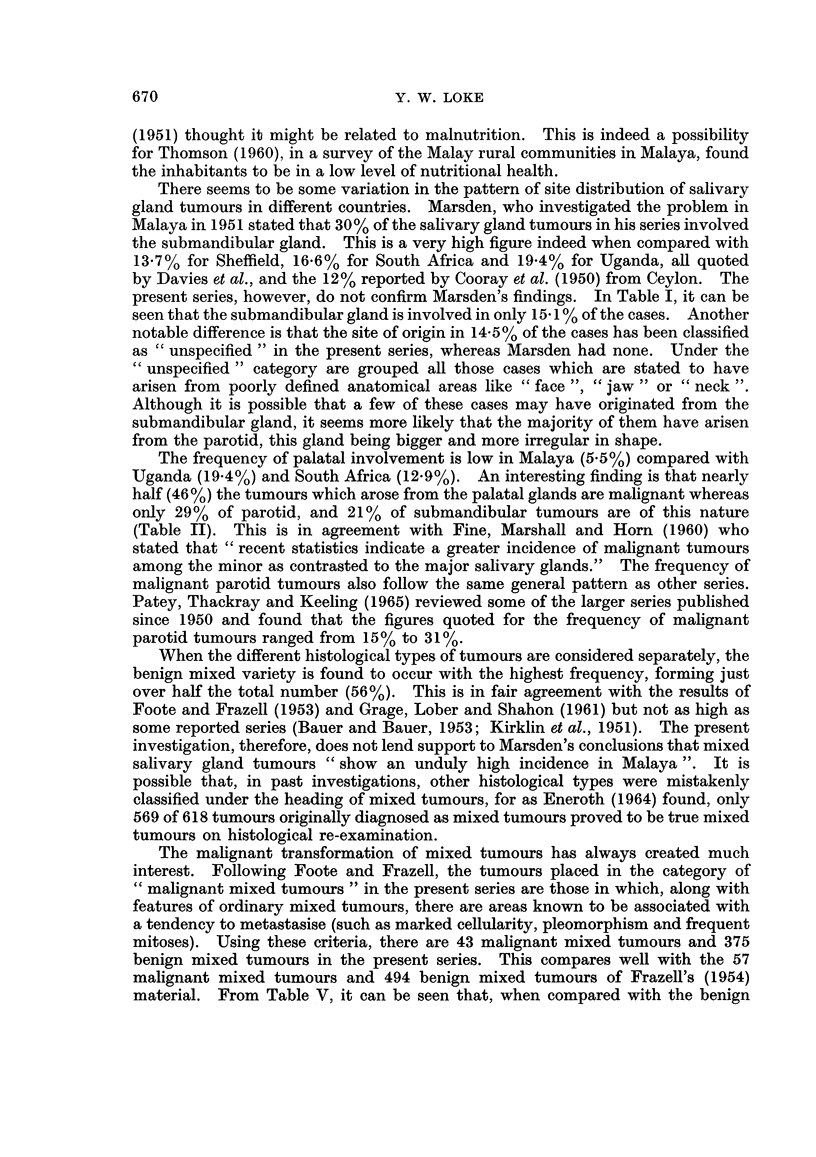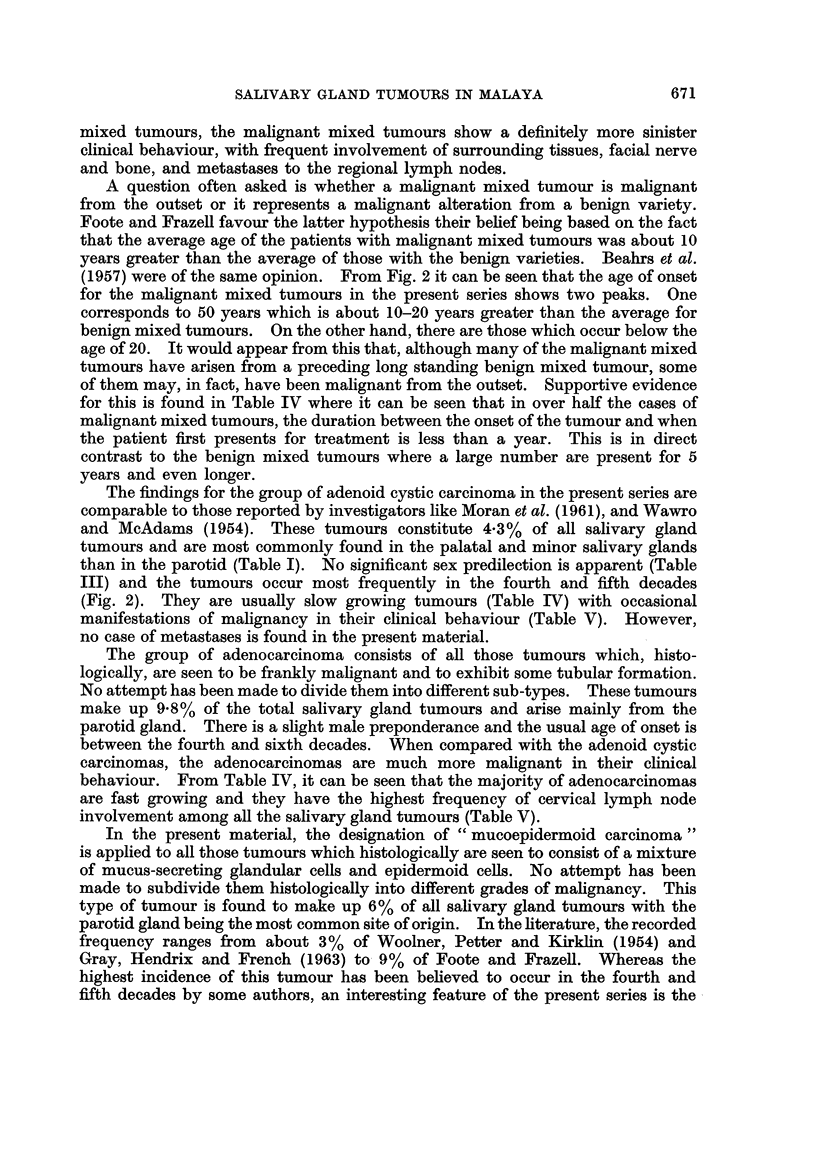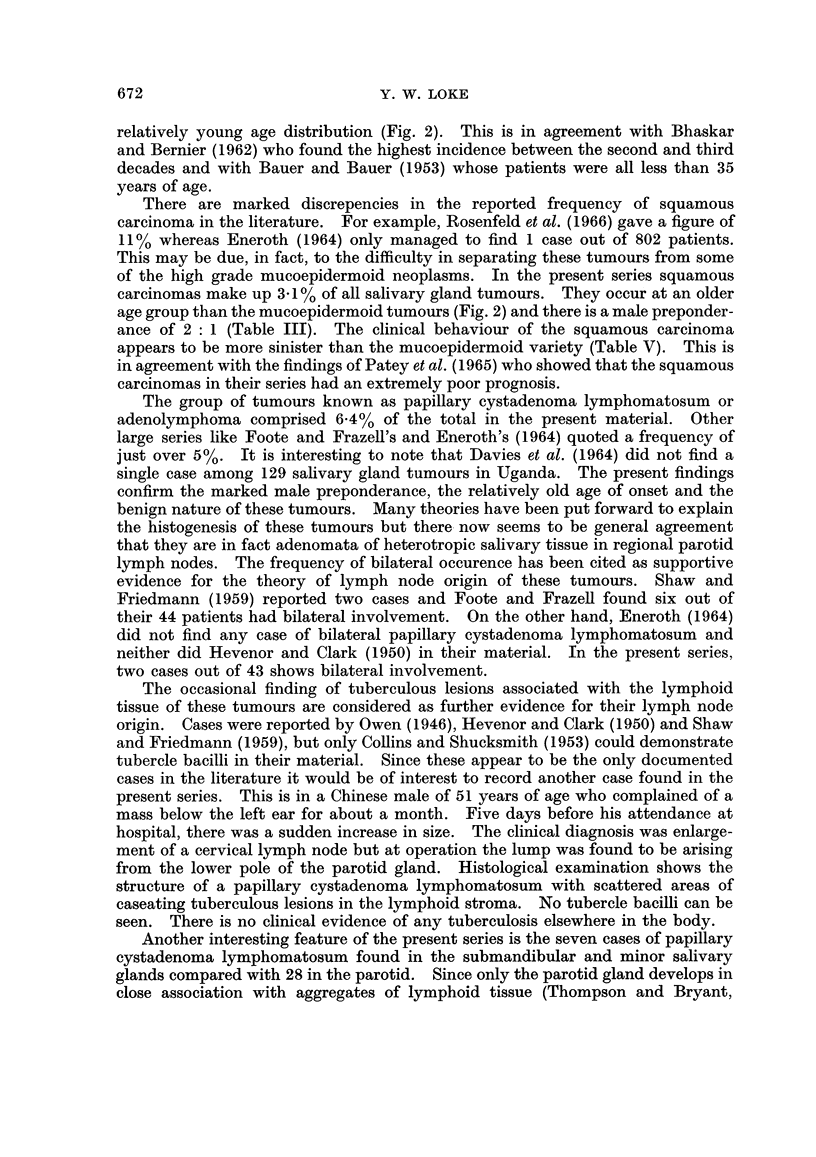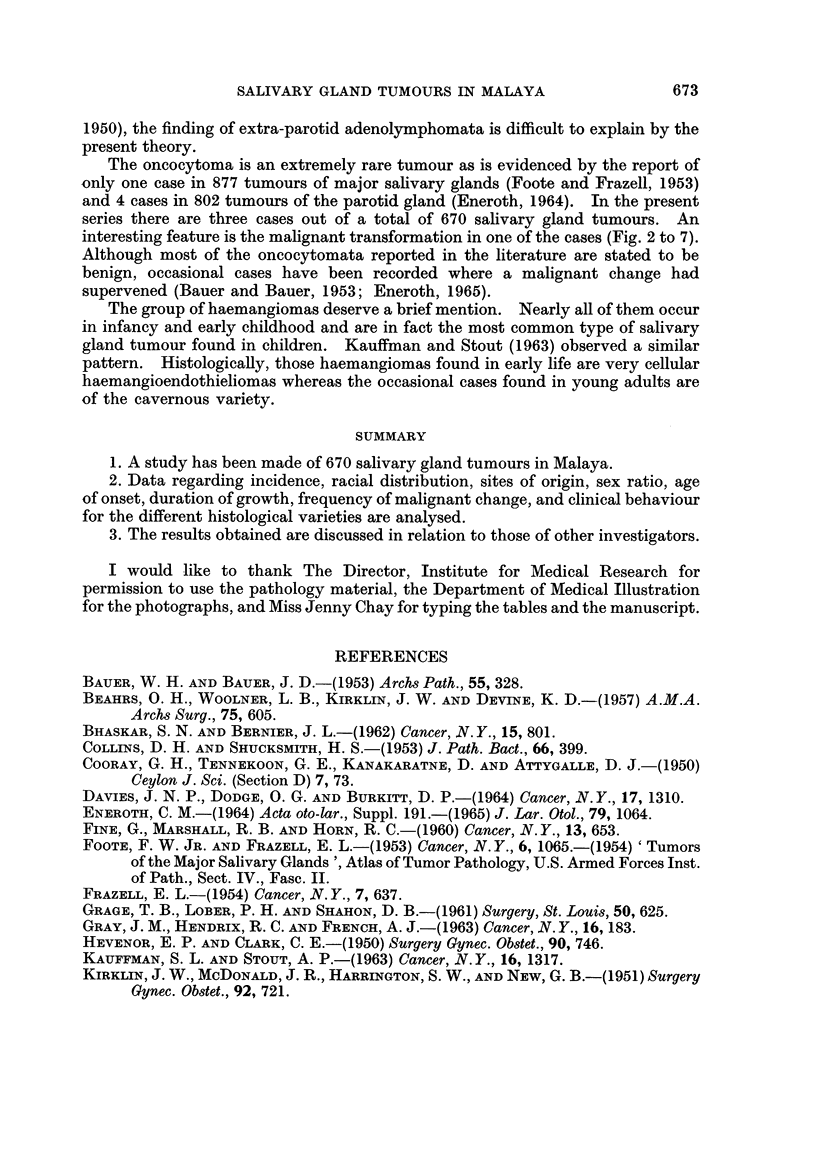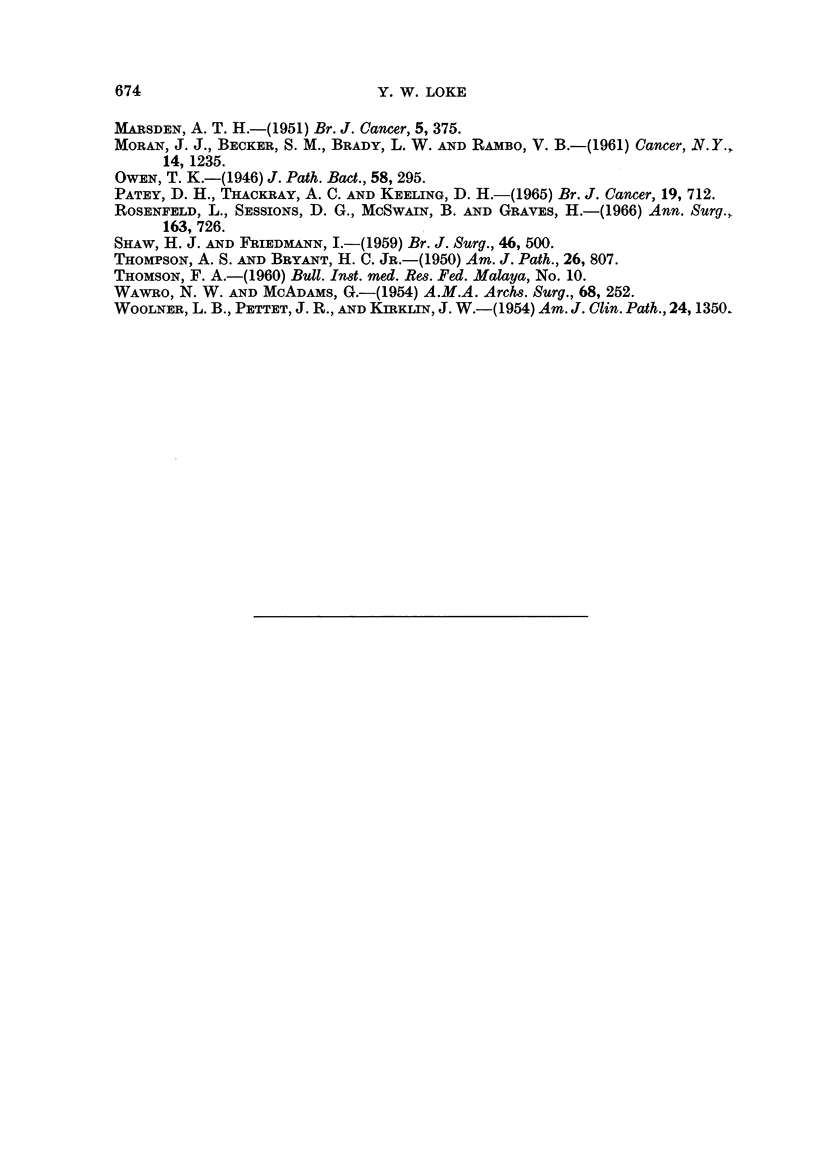# Salivary gland tumours in Malaya.

**DOI:** 10.1038/bjc.1967.78

**Published:** 1967-12

**Authors:** Y. W. Loke

## Abstract

**Images:**


### SALIVARY GLAND TUMOURS IN MALAYA

Y. W. LOKE*

*From the Department of Pathology, University of Malaya, Kuala Lumpur, Malaysia.*

Received for publication August 8, 1967

ALTHOUGH there is a voluminous amount of literature on salivary gland tumours, investigations based on significantly large number of cases have been relatively few. From this part of the world, reliable information is even less readily obtainable. In the present series, 670 salivary gland tumours are available for study. It is hoped that this large material may furnish additional data on these tumours and on those aspects which are peculiar to Malaya in particular.

### MATERIALS AND METHODS

This study is based on the material collected in the Pathology Division of the Institute for Medical Research, Kuala Lumpur, over the eighteen year period from 1948–1965 inclusive. The material comprised 670 cases of salivary gland tumours.

All histological sections were re-examined. Only the brief clinical histories which accompanied the specimens were available. These were studied in relation to the different histological types.

### RESULTS

### *Frequency*

The State of Selangor, which contains the capital of Malaya, has the best records and medical facilities. The standardised rate for salivary gland tumours computed in this state is therefore taken as a representative example.

In the year 1965, the end-of-year population figure is given as 694,064 males and 645,078 females in Selangor. During this year, 14 new cases of salivary gland tumours were seen, 9 of which were in males and 5 in females. This gives an incidence rate as follows:

Males: 1·3 per 100,000 population per year.
Females: 0·8 per 100,000 population per year.

### *Racial Distribution*

The population of Malaya is made up of three main racial groups: Malays, Chinese and Indians. The percentage of salivary gland tumours relative to the total number of tumours in these three races is shown in Fig. 1.

### *Histology*

The classification adopted is that of Foote and Frazell (1954). Table I shows the different histological varieties and their sites of origin.

* Present address: Department of Pathology, Tennis Court Road, University of Cambridge, England.

FIG. 1.—The percentage of salivary gland tumours relative to the total number of tumours in Malays, Chinese and Indians.

TABLE I—*Histological Types of Salivary Gland Tumours and their Sites of Origin*

| | Sites | | | | | | |
|---|---|---|---|---|---|---|---|
| Tumour type | Parotid | Submandibular | Palate | Minor | Unspecified | Total | % of total |
| Benign mixed tumour | 271 | 77 | 18 | 17 | 46 | 375 | 56 |
| Malignant mixed tumour | 24 | 4 | 5 | 1 | 9 | 43 | 6·4 |
| Adenoid cystic carcinoma | 9 | 0 | 6 | 10 | 4 | 29 | 4·3 |
| Adenocarcinoma | 37 | 5 | 5 | 9 | 10 | 66 | 9·8 |
| Papillary cystadenoma lymphomatosum | 28 | 5 | 1 | 1 | 5 | 43 | 6·4 |
| Mucoepidermoid carcinoma | 28 | 5 | 1 | 1 | 5 | 40 | 6·0 |
| Squamous carcinoma | 13 | 4 | 0 | 0 | 4 | 21 | 3·1 |
| Haemangioma | 20 | 0 | 0 | 2 | 1 | 23 | 3·4 |
| Oncocytoma | 2 | 1 | 0 | 0 | 0 | 3 | 0·45 |
| Adenoma | 2 | 0 | 2 | 0 | 1 | 5 | 0·75 |
| Neurofibroma | 8 | 2 | 0 | 1 | 1 | 12 | 1·8 |
| Lymphosarcoma | 0 | 2 | 0 | 0 | 0 | 2 | 0·3 |
| Metastatic melanoma | 1 | 0 | 0 | 0 | 0 | 1 | 0·15 |
| Undifferentiated malignant tumours | 4 | 1 | 0 | 0 | 2 | 7 | 1·0 |
| Total | 393 | 101 | 37 | 42 | 97 | 670 | |
| % of total | 58·7% | 15·1% | 5·5% | 6·3% | 14·5% | | |

A further subdivision of the figures shows that there is a higher frequency of malignant tumours arising from the palate and minor salivary glands than in the parotid and submandibular glands (Table II).

TABLE II—*Proportion of Benign and Malignant Salivary Gland Tumours in the Different Sites*

| Tumour type | Parotid | Submanibular | Palate | Minor |
|---|---|---|---|---|
| Benign tumours | 277 (71%) | 80 (79%) | 20 (54%) | 21 (50%) |
| Malignant tumours | 166 (29%) | 21 (21%) | 17 (46%) | 21 (50%) |
| Total | 393 | 101 | 37 | 42 |

The histological descriptions of the different types of tumours are well documented; it is, therefore, unnecessary to repeat them here. However, in the re-examination of the slides in the present series, one interesting case is seen which merits further comment. This is a malignant oncocytoma removed from the left parotid gland of a 70-year-old Chinese man. Histologically all stages of transition from a benign tumour to a frankly malignant one can be traced. In most parts the tumour shows the typical appearance of an oncocytoma, with cells of uniform size containing pink granular cytoplasm and a distinct vesicular nucleus (Fig. 2). Scattered among these cells, however, are small groups of cells which have a more bizarre appearance. The nuclei are very large and are irregular in shape and size. Abnormal mitotic figures can be seen (Fig. 3 and 4). In some parts large portions of the tumour are found to be replaced by these atypical cells (Fig. 5). In yet other areas the tumour has become frankly malignant, consisting of cells so undifferentiated and pleomorphic that all resemblance to salivary gland tissue is lost (Fig. 6). There is infiltration of the underlying bone (Fig. 7).

*Sex Distribution*

This is shown in Table III for the different histological types.

TABLE III.—*Sex Distribution for the Different Histological Types of Salivary Gland Tumours*

| | Male | | Female | |
|---|---|---|---|---|
| Tumour type | Number | Per cent | Number | Per cent |
| Benign mixed tumour | 192 | 51 | 182 | 49 |
| Malignant mixed tumour | 26 | 60·5 | 17 | 39·5 |
| Adenoid cystic carcinoma | 15 | 51·7 | 14 | 48·3 |
| Adenocarcinoma | 35 | 53 | 31 | 47 |
| Papillary cystadenoma lymphomatosum | 36 | 83·7 | 7 | 16·3 |
| Mucoepidermoid carcinoma | 23 | 57·5 | 17 | 42·5 |
| Squamous carcinoma | 14 | 66·7 | 7 | 33·3 |
| Haemangioma | 9 | 39 | 14 | 61 |
| Total | 350 | 54·8 | 289 | 45·2 |

*Age Distribution*

Since many of the salivary gland tumours are extremely slow growing, the age of onset of the tumour, rather than the age when the patient first presents, will give a more representative picture of the age distribution. The results are shown by the histogram in Fig. 8.

*Duration of Tumours*

The time interval between the onset of the tumour and when the patient first presents for treatment is given in Table IV.

TABLE IV.—*Relation of Duration of Tumour to Histological Type*

| | Duration in years | | | |
|---|---|---|---|---|
| | < 1 | 1–5 | 5–10 | > 10 |
| Benign mixed tumour | 67 | 159 | 31 | 28 |
| Malignant mixed tumour | 13 | 7 | 3 | 2 |
| Adenoid cystic carcinoma | 5 | 12 | 1 | 1 |
| Adenocarcinoma | 23 | 17 | 2 | 1 |
| Papillary cystadenoma lymphomatosum | 13 | 16 | 2 | 2 |
| Mucoepidermoid carcinoma | 9 | 12 | 5 | 0 |
| Squamous carcinoma | 10 | 4 | 0 | 2 |

*Clinical Aspects*

The invasive qualities of salivary gland tumours are usually manifested by ulceration of the overlying skin or mucous membrane, involvement of the facial nerve and bone, attachment to surrounding subcutaneous tissues, and metastases to the adjacent lymph nodes. The frequency with which these structures are involved by the different histological varieties of salivary gland tumours is given in Table V. No follow-up histories are available and none of the cases are examined

TABLE V.—*The Frequency of Involvement of Various Tissues by the Different Histological Types of Salivary Gland Tumours*

| | Frequency of involvement | | | | |
|---|---|---|---|---|---|
| Tumour type | Skin or mucous membrane | Facial nerve | Surround-ing tissue | Bone | Cervical lymph node |
| Benign mixed tumour | 1 | 2 | 2 | 0 | 0 |
| Malignant mixed tumour | 6 | 7 | 2 | 4 | 3 |
| Adenoid cystic carcinoma | 4 | 1 | 1 | 0 | 0 |
| Adenocarcinoma | 11 | 6 | 5 | 4 | 11 |
| Papillary cystadenoma lymphomatosum | 0 | 0 | 0 | 0 | 0 |
| Mucoepidermoid carcinoma | 0 | 1 | 3 | 0 | 2 |
| Squamous carcinoma | 3 | 2 | 3 | 1 | 4 |
| Haemangioma | 1 | 0 | 2 | 0 | 0 |
| Oncocytoma | 0 | 1 | 1 | 1 | 0 |
| Adenoma | 0 | 0 | 0 | 0 | 0 |
| Neurofibroma | 0 | 0 | 1 | 0 | 0 |
| Lymphosarcoma | 0 | 0 | 0 | 0 | 0 |
| Metastatic melanoma | 0 | 0 | 1 | 0 | 0 |
| Undifferentiated malignant tumour | 0 | 0 | 2 | 0 | 0 |
| Total | 26 | 20 | 23 | 10 | 20 |

EXPLANATION OF PLATES

FIG. 2.—Typical appearance of an oncocytoma. H. & E. ×350.
FIG. 3.—Occasional large, bizarre looking cells. H. & E. ×520.
FIG. 4.—Large cell with abnormal mitotic figure. H. & E. ×520.
FIG. 5.—Large areas of the oncocytoma replaced by abnormal looking cells. H. & E. ×350.
FIG. 6.—Frankly malignant area showing no resemblance to salivary gland tissue. H. & E. ×350.
FIG. 7.—Tumour invading bone. H. & E. ×350.

2

3

Loke.

4

5

6

7

Loke.

FIG. 8.—Age of onset for the different histological types of salivary gland tumours, shown as numbers of cases in each decade.

post-mortem so it is not possible to comment on the frequency of metastases to other organs, but at the time of presentation no clinical evidence of distant metastases has been observed in any of the cases.

### DISCUSSION

It has often been said that salivary gland tumours are unduly common in the poor, tropical areas of the world. Davies, Dodge and Burkitt (1964), however, found no evidence to support this popular belief. Their standardised rates for Africans in the district of Kyadondo were no higher when compared with figures for the United States and Norway. The present findings in Malaya of 1·3 and 0·8 per 100,000 population per year for males and females respectively are comparable to those quoted by Davies *et al.*, except that the male : female ratio appears to be reversed.

When the frequency of salivary gland tumours is viewed in relation to the total number of tumours, the figure of 4·1% obtained for the Malays is relatively high compared with the 2·3% for the Chinese and 1·7% for the Indians (Fig. 1). In European races, salivary gland tumours comprise less than 3% of all tumours (Eneroth, 1964). The Malay preponderance is difficult to explain but Marsden

(1951) thought it might be related to malnutrition. This is indeed a possibility for Thomson (1960), in a survey of the Malay rural communities in Malaya, found the inhabitants to be in a low level of nutritional health.

There seems to be some variation in the pattern of site distribution of salivary gland tumours in different countries. Marsden, who investigated the problem in Malaya in 1951 stated that 30% of the salivary gland tumours in his series involved the submandibular gland. This is a very high figure indeed when compared with 13·7% for Sheffield, 16·6% for South Africa and 19·4% for Uganda, all quoted by Davies *et al.*, and the 12% reported by Cooray *et al.* (1950) from Ceylon. The present series, however, do not confirm Marsden's findings. In Table I, it can be seen that the submandibular gland is involved in only 15·1% of the cases. Another notable difference is that the site of origin in 14·5% of the cases has been classified as " unspecified " in the present series, whereas Marsden had none. Under the " unspecified " category are grouped all those cases which are stated to have arisen from poorly defined anatomical areas like " face ", " jaw " or " neck ". Although it is possible that a few of these cases may have originated from the submandibular gland, it seems more likely that the majority of them have arisen from the parotid, this gland being bigger and more irregular in shape.

The frequency of palatal involvement is low in Malaya (5·5%) compared with Uganda (19·4%) and South Africa (12·9%). An interesting finding is that nearly half (46%) the tumours which arose from the palatal glands are malignant whereas only 29% of parotid, and 21% of submandibular tumours are of this nature (Table II). This is in agreement with Fine, Marshall and Horn (1960) who stated that " recent statistics indicate a greater incidence of malignant tumours among the minor as contrasted to the major salivary glands." The frequency of malignant parotid tumours also follow the same general pattern as other series. Patey, Thackray and Keeling (1965) reviewed some of the larger series published since 1950 and found that the figures quoted for the frequency of malignant parotid tumours ranged from 15% to 31%.

When the different histological types of tumours are considered separately, the benign mixed variety is found to occur with the highest frequency, forming just over half the total number (56%). This is in fair agreement with the results of Foote and Frazell (1953) and Grage, Lober and Shahon (1961) but not as high as some reported series (Bauer and Bauer, 1953; Kirklin *et al.*, 1951). The present investigation, therefore, does not lend support to Marsden's conclusions that mixed salivary gland tumours " show an unduly high incidence in Malaya ". It is possible that, in past investigations, other histological types were mistakenly classified under the heading of mixed tumours, for as Eneroth (1964) found, only 569 of 618 tumours originally diagnosed as mixed tumours proved to be true mixed tumours on histological re-examination.

The malignant transformation of mixed tumours has always created much interest. Following Foote and Frazell, the tumours placed in the category of " malignant mixed tumours " in the present series are those in which, along with features of ordinary mixed tumours, there are areas known to be associated with a tendency to metastasise (such as marked cellularity, pleomorphism and frequent mitoses). Using these criteria, there are 43 malignant mixed tumours and 375 benign mixed tumours in the present series. This compares well with the 57 malignant mixed tumours and 494 benign mixed tumours of Frazell's (1954) material. From Table V, it can be seen that, when compared with the benign mixed tumours, the malignant mixed tumours show a definitely more sinister clinical behaviour, with frequent involvement of surrounding tissues, facial nerve and bone, and metastases to the regional lymph nodes.

A question often asked is whether a malignant mixed tumour is malignant from the outset or it represents a malignant alteration from a benign variety. Foote and Frazell favour the latter hypothesis their belief being based on the fact that the average age of the patients with malignant mixed tumours was about 10 years greater than the average of those with the benign varieties. Beahrs *et al.* (1957) were of the same opinion. From Fig. 2 it can be seen that the age of onset for the malignant mixed tumours in the present series shows two peaks. One corresponds to 50 years which is about 10–20 years greater than the average for benign mixed tumours. On the other hand, there are those which occur below the age of 20. It would appear from this that, although many of the malignant mixed tumours have arisen from a preceding long standing benign mixed tumour, some of them may, in fact, have been malignant from the outset. Supportive evidence for this is found in Table IV where it can be seen that in over half the cases of malignant mixed tumours, the duration between the onset of the tumour and when the patient first presents for treatment is less than a year. This is in direct contrast to the benign mixed tumours where a large number are present for 5 years and even longer.

The findings for the group of adenoid cystic carcinoma in the present series are comparable to those reported by investigators like Moran *et al.* (1961), and Wawro and McAdams (1954). These tumours constitute 4·3% of all salivary gland tumours and are most commonly found in the palatal and minor salivary glands than in the parotid (Table I). No significant sex predilection is apparent (Table III) and the tumours occur most frequently in the fourth and fifth decades (Fig. 2). They are usually slow growing tumours (Table IV) with occasional manifestations of malignancy in their clinical behaviour (Table V). However, no case of metastases is found in the present material.

The group of adenocarcinoma consists of all those tumours which, histologically, are seen to be frankly malignant and to exhibit some tubular formation. No attempt has been made to divide them into different sub-types. These tumours make up 9·8% of the total salivary gland tumours and arise mainly from the parotid gland. There is a slight male preponderance and the usual age of onset is between the fourth and sixth decades. When compared with the adenoid cystic carcinomas, the adenocarcinomas are much more malignant in their clinical behaviour. From Table IV, it can be seen that the majority of adenocarcinomas are fast growing and they have the highest frequency of cervical lymph node involvement among all the salivary gland tumours (Table V).

In the present material, the designation of " mucoepidermoid carcinoma " is applied to all those tumours which histologically are seen to consist of a mixture of mucus-secreting glandular cells and epidermoid cells. No attempt has been made to subdivide them histologically into different grades of malignancy. This type of tumour is found to make up 6% of all salivary gland tumours with the parotid gland being the most common site of origin. In the literature, the recorded frequency ranges from about 3% of Woolner, Petter and Kirklin (1954) and Gray, Hendrix and French (1963) to 9% of Foote and Frazell. Whereas the highest incidence of this tumour has been believed to occur in the fourth and fifth decades by some authors, an interesting feature of the present series is the relatively young age distribution (Fig. 2). This is in agreement with Bhaskar and Bernier (1962) who found the highest incidence between the second and third decades and with Bauer and Bauer (1953) whose patients were all less than 35 years of age.

There are marked discrepencies in the reported frequency of squamous carcinoma in the literature. For example, Rosenfeld *et al.* (1966) gave a figure of 11% whereas Eneroth (1964) only managed to find 1 case out of 802 patients. This may be due, in fact, to the difficulty in separating these tumours from some of the high grade mucoepidermoid neoplasms. In the present series squamous carcinomas make up 3·1% of all salivary gland tumours. They occur at an older age group than the mucoepidermoid tumours (Fig. 2) and there is a male preponderance of 2 : 1 (Table III). The clinical behaviour of the squamous carcinoma appears to be more sinister than the mucoepidermoid variety (Table V). This is in agreement with the findings of Patey *et al.* (1965) who showed that the squamous carcinomas in their series had an extremely poor prognosis.

The group of tumours known as papillary cystadenoma lymphomatosum or adenolymphoma comprised 6·4% of the total in the present material. Other large series like Foote and Frazell's and Eneroth's (1964) quoted a frequency of just over 5%. It is interesting to note that Davies *et al.* (1964) did not find a single case among 129 salivary gland tumours in Uganda. The present findings confirm the marked male preponderance, the relatively old age of onset and the benign nature of these tumours. Many theories have been put forward to explain the histogenesis of these tumours but there now seems to be general agreement that they are in fact adenomata of heterotropic salivary tissue in regional parotid lymph nodes. The frequency of bilateral occurence has been cited as supportive evidence for the theory of lymph node origin of these tumours. Shaw and Friedmann (1959) reported two cases and Foote and Frazell found six out of their 44 patients had bilateral involvement. On the other hand, Eneroth (1964) did not find any case of bilateral papillary cystadenoma lymphomatosum and neither did Hevenor and Clark (1950) in their material. In the present series, two cases out of 43 shows bilateral involvement.

The occasional finding of tuberculous lesions associated with the lymphoid tissue of these tumours are considered as further evidence for their lymph node origin. Cases were reported by Owen (1946), Hevenor and Clark (1950) and Shaw and Friedmann (1959), but only Collins and Shucksmith (1953) could demonstrate tubercle bacilli in their material. Since these appear to be the only documented cases in the literature it would be of interest to record another case found in the present series. This is in a Chinese male of 51 years of age who complained of a mass below the left ear for about a month. Five days before his attendance at hospital, there was a sudden increase in size. The clinical diagnosis was enlargement of a cervical lymph node but at operation the lump was found to be arising from the lower pole of the parotid gland. Histological examination shows the structure of a papillary cystadenoma lymphomatosum with scattered areas of caseating tuberculous lesions in the lymphoid stroma. No tubercle bacilli can be seen. There is no clinical evidence of any tuberculosis elsewhere in the body.

Another interesting feature of the present series is the seven cases of papillary cystadenoma lymphomatosum found in the submandibular and minor salivary glands compared with 28 in the parotid. Since only the parotid gland develops in close association with aggregates of lymphoid tissue (Thompson and Bryant,

1950), the finding of extra-parotid adenolymphomata is difficult to explain by the present theory.

The oncocytoma is an extremely rare tumour as is evidenced by the report of only one case in 877 tumours of major salivary glands (Foote and Frazell, 1953) and 4 cases in 802 tumours of the parotid gland (Eneroth, 1964). In the present series there are three cases out of a total of 670 salivary gland tumours. An interesting feature is the malignant transformation in one of the cases (Fig. 2 to 7). Although most of the oncocytomata reported in the literature are stated to be benign, occasional cases have been recorded where a malignant change had supervened (Bauer and Bauer, 1953; Eneroth, 1965).

The group of haemangiomas deserve a brief mention. Nearly all of them occur in infancy and early childhood and are in fact the most common type of salivary gland tumour found in children. Kauffman and Stout (1963) observed a similar pattern. Histologically, those haemangiomas found in early life are very cellular haemangioendothieliomas whereas the occasional cases found in young adults are of the cavernous variety.

SUMMARY

1. A study has been made of 670 salivary gland tumours in Malaya.
2. Data regarding incidence, racial distribution, sites of origin, sex ratio, age of onset, duration of growth, frequency of malignant change, and clinical behaviour for the different histological varieties are analysed.
3. The results obtained are discussed in relation to those of other investigators.

I would like to thank The Director, Institute for Medical Research for permission to use the pathology material, the Department of Medical Illustration for the photographs, and Miss Jenny Chay for typing the tables and the manuscript.

### REFERENCES

Bauer, W. H. and Bauer, J. D.—(1953) *Archs Path.*, **55**, 328.

Beahrs, O. H., Woolner, L. B., Kirklin, J. W. and Devine, K. D.—(1957) *A.M.A. Archs Surg.*, **75**, 605.

Bhaskar, S. N. and Bernier, J. L.—(1962) *Cancer, N.Y.*, **15**, 801.

Collins, D. H. and Shucksmith, H. S.—(1953) *J. Path. Bact.*, **66**, 399.

Cooray, G. H., Tennekoon, G. E., Kanakaratne, D. and Attygalle, D. J.—(1950) *Ceylon J. Sci.* (Section D) **7**, 73.

Davies, J. N. P., Dodge, O. G. and Burkitt, D. P.—(1964) *Cancer, N.Y.*, **17**, 1310.

Eneroth, C. M.—(1964) *Acta oto-lar.*, Suppl. 191.—(1965) *J. Lar. Otol.*, **79**, 1064.

Fine, G., Marshall, R. B. and Horn, R. C.—(1960) *Cancer, N.Y.*, **13**, 653.

Foote, F. W. Jr. and Frazell, E. L.—(1953) *Cancer, N.Y.*, **6**, 1065.—(1954) ' Tumors of the Major Salivary Glands ', Atlas of Tumor Pathology, U.S. Armed Forces Inst. of Path., Sect. IV., Fasc. II.

Frazell, E. L.—(1954) *Cancer, N.Y.*, **7**, 637.

Grage, T. B., Lober, P. H. and Shahon, D. B.—(1961) *Surgery, St. Louis*, **50**, 625.

Gray, J. M., Hendrix, R. C. and French, A. J.—(1963) *Cancer, N.Y.*, **16**, 183.

Hevenor, E. P. and Clark, C. E.—(1950) *Surgery Gynec. Obstet.*, **90**, 746.

Kauffman, S. L. and Stout, A. P.—(1963) *Cancer, N.Y.*, **16**, 1317.

Kirklin, J. W., McDonald, J. R., Harrington, S. W., and New, G. B.—(1951) *Surgery Gynec. Obstet.*, **92**, 721.

MARSDEN, A. T. H.—(1951) *Br. J. Cancer*, **5**, 375.
MORAN, J. J., BECKER, S. M., BRADY, L. W. AND RAMBO, V. B.—(1961) *Cancer, N.Y.*, **14**, 1235.
OWEN, T. K.—(1946) *J. Path. Bact.*, **58**, 295.
PATEY, D. H., THACKRAY, A. C. AND KEELING, D. H.—(1965) *Br. J. Cancer*, **19**, 712.
ROSENFELD, L., SESSIONS, D. G., McSWAIN, B. AND GRAVES, H.—(1966) *Ann. Surg.*, **163**, 726.
SHAW, H. J. AND FRIEDMANN, I.—(1959) *Br. J. Surg.*, **46**, 500.
THOMPSON, A. S. AND BRYANT, H. C. JR.—(1950) *Am. J. Path.*, **26**, 807.
THOMSON, F. A.—(1960) *Bull. Inst. med. Res. Fed. Malaya*, No. 10.
WAWRO, N. W. AND McADAMS, G.—(1954) *A.M.A. Archs. Surg.*, **68**, 252.
WOOLNER, L. B., PETTET, J. R., AND KIRKLIN, J. W.—(1954) *Am. J. Clin. Path.*, **24**, 1350.